# ^18^F-FAPI-04 PET/CT parameters predict PD-L1 expression in esophageal squamous cell carcinoma

**DOI:** 10.3389/fimmu.2023.1266843

**Published:** 2023-11-15

**Authors:** Yaqing Zhao, Jiazhong Ren

**Affiliations:** ^1^ Department of General Affairs Section, The Second Affiliated Hospital of Shandong University of Traditional Chinese Medicine, Jinan, Shandong, China; ^2^ Department of Medical Imaging, PET-CT Center, Shandong Cancer Hospital and Institute, Shandong First Medical University and Shandong Academy of Medical Sciences, Jinan, Shandong, China

**Keywords:** fibroblast activation protein, positron emission tomography, PD-L1 expression, esophageal carcinoma, 18F-FAPI-04 PET/CT parameters

## Abstract

**Purpose:**

This prospective study examined whether metabolism parameters obtained using the tracer ^18^F-AlFNOTA-fibroblast activation protein inhibitor (FAPI)-04 (denoted as ^18^F-FAPI-04) in positron emission tomography/computed tomography (PET/CT) can predict programmed death ligand-1 (PD-L1) expression in patients with locally advanced esophageal squamous cell carcinoma (LA-ESCC).

**Patients and methods:**

The 24 enrolled LA-ESCC patients underwent an ^18^F-FAPI-04 PET/CT scan. The maximum, mean, peak and standard deviation standard uptake values (SUVmax, SUVmean, SUVpeak and SUVsd), metabolic tumor volume (MTV), and total lesion FAP (TLF) expression of the primary tumor were collected. Additionally, we evaluated PD-L1 expression on cancer cells by immunohistochemistry and immunofluorescence methods. Patients were divided into negative and positive expressions according to the expression of PD-L1 (CPS < 10 and CPS ≥ 10), and the variables were compared between the two groups.

**Results:**

The SUVmax, SUVmean, SUVpeak and SUVsd were significantly higher in patients with positive expression than in negative expression (all p < 0.05). Receiver operating characteristic curve analysis identified SUVmean (area under the curve [AUC] = 0.882, p = 0.004), SUVsd (AUC = 0.874, p = 0.005), SUVpeak (AUC = 0.840, p = 0.010) and SUVmax (AUC = 0.765, p = 0.045) as significant predictors of the PD-L1 positive expression, with cutoff values of 9.67, 1.90, 9.67 and 13.71, respectively. On univariate logistic regression analysis, SUVmean (p = 0.045), SUVsd (p = 0.024), and SUVpeak (p = 0.031) were significantly correlated with the PD-L1 positive expression. On multivariable logistic regression analysis, SUVsd (p = 0.035) was an optimum predictor factor for PD-L1 positive expression.

**Conclusion:**

^18^F-FAPI-04 PET/CT parameters, including SUVmean, SUVpeak, and SUVsd, correlated with PD-L1 expression in patients with LA-ESCC, and thus SUVsd was an optimum predictor for PD-L1 positive expression, which could help to explore the existence of immune checkpoints and select ESCC candidates for immunotherapy.

## 1 Introduction

Esophageal cancer (EC) is one of the most common malignant tumors of the digestive system in the world, ranking seventh in incidence and sixth in mortality overall in 2020 (1, 2). Esophageal squamous cell carcinoma (ESCC) is the main histological type of esophageal cancer in China. The prognosis for esophageal cancer is poor, with a 5-year survival rate of only 15%-25% worldwide (3). At present, the treatment options available are limited.

With the development and application of immunotherapy, programmed death ligand-1 (PD-L1) has been shown to significantly prolong the overall survival of EC patients with manageable safety (4–6). In 2019, the immune checkpoint inhibitor pembrolizumab was approved by the Food and Drug Administration (FDA) as a second-line therapy to treat patients with locally advanced or metastatic ESCC whose tumors are positively expressing PD-L1 (Combined Positive Score [CPS] ≥10) (5). Immunohistochemistry (IHC) expression of PD-L1 is the most widely used biomarker for predicting the efficacy of esophageal cancer immunotherapy, and accurate and reliable PD-L1 testing is crucial for screening potential beneficiaries of immunotherapy.

Fibroblast activation protein (FAP) is a member of the dipeptidyl peptidase 4 protein family and has both endopeptidase and dipeptidyl peptidase activities. FAP is highly expressed in stromal fibroblasts of more than 90% of epithelial carcinomas (7, 8). Research has shown that high expression of FAP in stromal fibroblasts of breast cancer, colon cancer, esophageal cancer and other malignant tumors is related to poor prognosis (9–11). ^68^Ga-DOTAFAPI-04 has diagnostic and therapeutic potential in oncologic and nononcologic diseases (12, 13). ^68^Ga-FAPI-04 has been explored the value of predicting treatment outcomes and prognosis for EC patients (14–16). We previously performed a pilot clinical study in which ^18^F-FAPI-04, a novel tracer, was safe and offered high specificity for FAP imaging (17). However, the ability of ^68^Ga-FAPI/^18^F-FAPI-04 PET/CT to predict PD-L1 expression in EC needs to be validated by prospective studies.

The present study aimed to identify imaging parameters that could predict tumor PD-L1 expression by comparing ^18^F-FAPI-04 PET/CT parameters between patients with EC classified as negative (CPS < 10) and positive expression (CPS ≥ 10). Identifying patients with PD-L1 positively expressed by imaging will help realize the individualized treatment of tumors and improve prognosis.

## 2 Methods

### 2.1 Patients

Potentially eligible locally advanced esophageal squamous cell carcinoma (LA-ESCC) patients were recruited at Shandong Cancer Hospital and Institute from June 2021 to July 2022 (**Table 1**). All patients volunteered to participate in this study, and the local ethics committee of Shandong Cancer Hospital and Institute approved the prospective study.

**Table 1:** Characteristics of enrolled LA-ESCC patients (N=24).

Characteristics		Number of cases (%)
Age (years)	≤60	11 (45.8)
	>60	13 (54.2)
Gender	Male	21 (87.5)
	Female	3 (12.5)
T stage	T3	24 (100.0)
	T4	0 (0.0)
N stage	N0	5 (20.8)
	N1	14 (58.3)
	N2	3 (12.5)
	N3	2 (8.3)
Tumor location	Cervical	0 (0.0)
	Upper	7 (29.2)
	Middle	10 (41.7)
	Lower	7 (29.2)

Patients were enrolled based on the following criteria: (1) histopathologically confirmed esophageal squamous cell carcinoma(T3~4N0~3M0); (2) age ≥ 18 years; (3) presence of measurable primary tumors;(4) PD-L1 expression assay was conducted and (5) ^18^F-FAPI-04 PET/CT scanning was performed.

**Figure 1:**
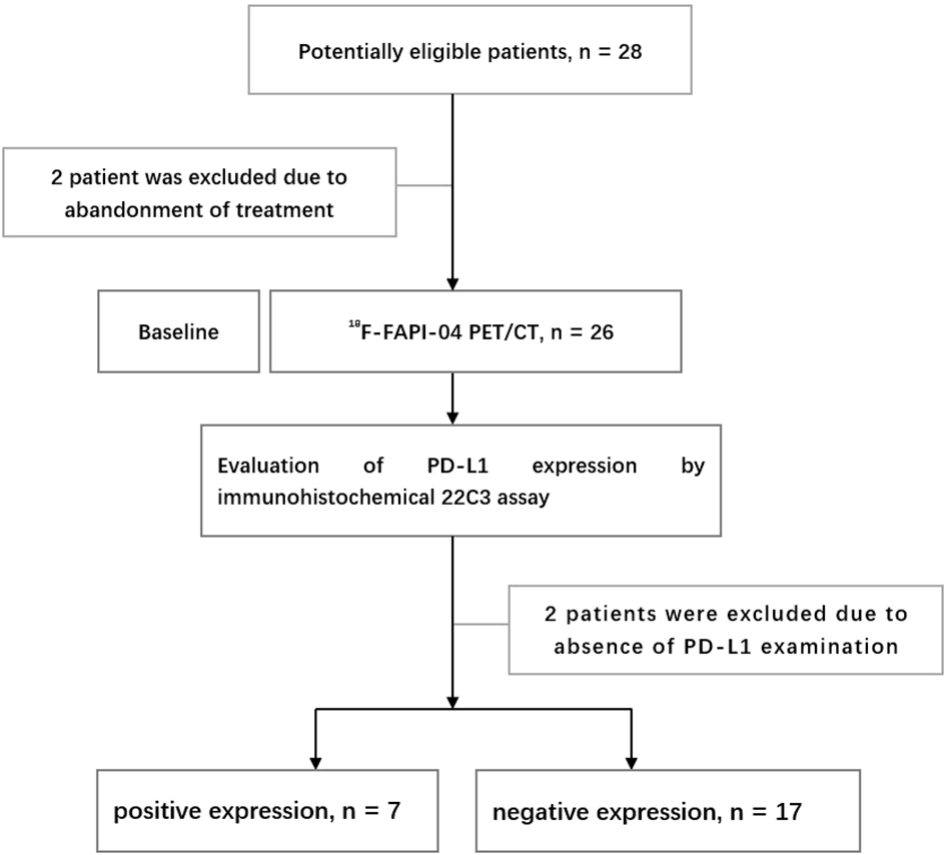
Research flowchart.

The exclusion criteria included: (1) pregnancy or breastfeeding; and (2) unwillingness to participate or withdraw. The flow chart of research and design is shown in **Figure 1**.

### 2.2 ^18^F-FAPI-04 PET/CT scanning

^18^F-FAPI-04 was synthesized as described previously (18). Patients were not required to fast or under blood glucose measurement before scanning. After intravenous injection of ^18^F-FAPI-04 (4.81 MBq/kg), the patients needed to rest for approximately 1 h. Scanning was then performed with two different PET/CT(GE MINI TF Big Bore; Philips Healthcare, Cleveland, OH, USA). Whole-body CT scans were obtained using a low-dose protocol (300 mAs, 120 kV, a 512 × 512 matrix, rotation time of 1.0 s, and pitch index of 0.688; reconstructed with a soft-tissue kernel to a slice thickness of 2 mm) for attenuation correction. PET data were acquired in three-dimensional mode using a 200 × 200 matrix with an imaging time of 1 min per bed position. During image acquisition, the patients maintained normal shallow breathing. Subsequently, after attenuation and correction (Biograph 3D iterative reconstruction software, time of flight [TOF] correction), we viewed attenuation-corrected PET images, CT images, and PET/CT fusion images.

### 2.3 Imaging analysis

The attenuation-corrected CT images, PET images, and fused PET/CT images were displayed in coronal, sagittal, and transaxial slices, which were viewed and analyzed on the Nuclear Medicine Information System (Beijing Mozi Healthcare Ltd, Beijing, China). Two experienced PET/CT physicians (J.Z. and J.R., with 18 and 6 years, respectively, of nuclear oncology experience) visually assessed the ^18^F-FAPI-04 PET/CT images and reached a consensus regarding the image interpretations for primary tumors. Regions of interest were drawn around tumor lesions with higher uptake in transaxial sections, and ^18^F-FAPI-04 PET/CT parameters were generated by an automated 3-dimensional contouring program with a 30% isocontour. The uptake values in the region of interest were normalised to the injected dose per kilogram of patient body weight, and the standardised uptake values were derived according to the following formula: [measured radioactivity concentration (Bq/mL) × body weight (g)]/injected radioactivity concentration (Bq). Regions of interest were drawn around the primary tumor lesion, and the obtained parameters, including SUVmax, SUVmean, SUVpeak, SUVsd, metabolic tumor volume (MTV), and total lesion FAP expression (TLF), were generated by an automated contouring program provided by the vendor. TLF (total lesion FAP expression) was calculated as the product of the SUVmean of the lesion and the MTV (TLF= SUVmean× MTV). We also measured the SUVmean of 1 cm^3^ areas in the ascending aorta, liver and Lumbar 5 (L5) vertebrae. The circular region of interest (ROI) of 1cm3 was drawn in the normal regions of segments VII and VIII of the liver. The average of the liver SUVmean was calculated. The ratio of the SUVmax of the primary tumor to the SUVmean of the normal tissue (blood, liver and L5 vertebrae) is then calculated and is called the tumor to background ratio (TBRblood, TBRliver and TBRbone). For controversial lesions, discussion among the imaging experts with consideration of the results from other imaging modalities proceeded until a final consensus was reached.

### 2.4 Evaluation of PD-L1 expression by immunohistochemical 22C3 assay

In our study, PD-L1 expression in all patients was obtained by gastroscopic biopsy for pathological tissue of esophageal cancer.

PD-L1 expression was assessed by CPS, which was defined as the number of PD-L1 stained cells (tumor cells, lymphocytes and macrophages) divided by the total number of surviving tumor cells multiplied by 100. The maximum CPS is defined as 100. All other cells, such as tumor-associated plasma cells, neutrophils, normal/non-neoplastic cells, and necrotic cells, were excluded from the evaluation. The cutoff value was determined according to an FDA-approved test and the guidelines of pembrolizumab treatment and separated into two classifications: negative (CPS < 10) and positive expression (CPS ≥ 10) (5, 17). Patients without sufficient viable tumor cells (<100) were excluded. Each slide was blindly given a CPS for PD-L1 expression by two experienced pathologists. Both hematoxylin–eosin (HE) staining and PD-L1 IHC staining were assessed to reach a final CPS value. Two experienced pathologists (D.Z. and H.J., with 25 and 22 years, respectively, of oncology experience) evaluated pathological slides. Each case has a final consistent result after discussion.

### 2.5 Statistical analysis

Statistical analyzes were performed using SPSS software (version 27.0 for Windows; SPSS INC.). Continuous data were described as the mean ± standard deviation (mean ± SD) or median and interquartile, depending on whether they followed a normal distribution. and non-normally distributed data (including MTV) was expressed as the median and interquartile. Comparisons of normally distributed data between the two groups were performed using a paired two-sample t test, and comparisons of non-normally distributed data between the two groups were performed using the Mann–Whitney U test. Binary logistic regression analyses were performed to ascertain the relationships between ^18^F-FAPI-04 PET/CT parameters, tumor location, degree of differentiation and PD-L1 expression. Receiver operating characteristic (ROC) curve analysis was used to determine the threshold values with the maximum Youden index of ^18^F-FAPI-04 PET/CT parameters for PD-L1 positive expression. Spearman rank correlation coefficients were calculated to assess the relationship between ^18^F-FAPI-04 PET/CT parameters and PD-L1 expression. All tests were two-sided, and a probability of less than 0.05 was considered statistically significant.

## 3 Results

### 3.1 Patients’ characteristics

**Figure 2:**
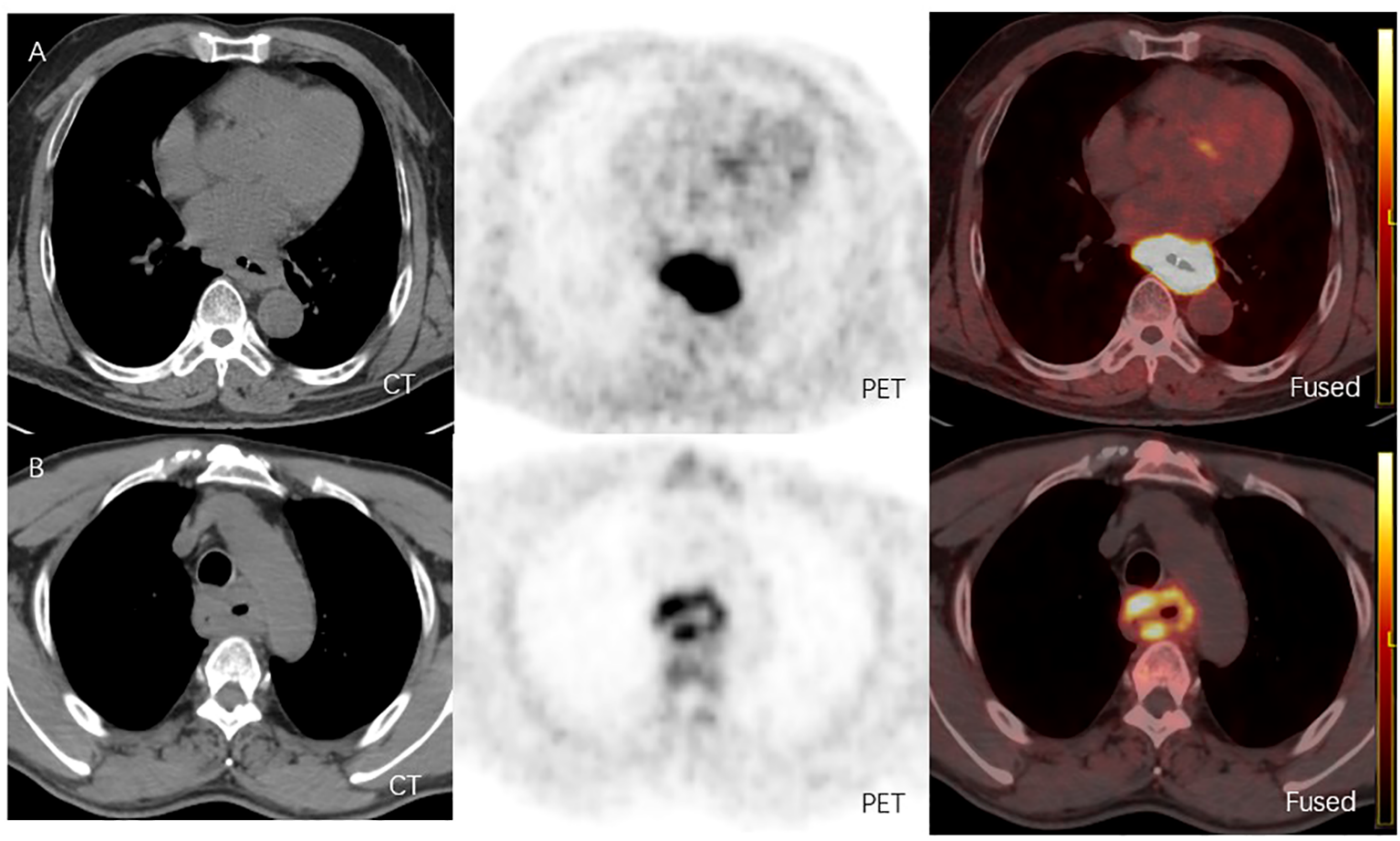
**(A)** 18F-FAPI-04 PET/CT and CT images of a LA-ESCC patient with an outcome classified as PD-L1 positive expression(CPS about 70), with SUVmax 15.36, SUVmean 8.60, SUVpeak 11.02, SUVsd 2.47, MTV 51.52 cm3 and TLF 443.07g. **(B)** 18F-FAPI-04 PET/CT and CT images of a LA-ESCC patient with an outcome classified as PD-L1 positive expression(CPS<1) , with SUVmax 6.45, SUVmean 3.36, SUVpeak 4.51, SUVsd 0.89, MTV 53.99 cm3 and TLF 181.41g.

From June 2021 to July 2022, 24 patients diagnosed with LA-ESCC based on histological examinations at Shandong Cancer Hospital and Institute were enrolled in this study. The characteristics of the patients are presented in **Table 1**. Among all patients, 17 patients were classified as negative expression (CPS < 10), and 7 patients as positive expression (CPS ≥ 10). **Figure 2** shows representative ^18^F-FAPI-04 PET/CT imaging results for two cases classified as positive and negative PD-L1 expression.

### 3.2 Quantitative ^18^F-FAPI-04 PET/CT parameters

The quantitative ^18^F-FAPI-04 PET/CT parameters SUVmax, SUVmean, SUVpeak, SUVsd, MTV and TLF are shown in **Table 2** for all patients, negative (CPS < 10) and positive expression (CPS≥10) patients. SUVmax, SUVmean, SUVpeak, and SUVsd were significantly higher in positive expression patients than in negative (14.13 ± 4.41 vs. 10.61 ± 2.77, p = 0.027; 8.67 ± 1.97 vs. 5.74 ± 1.60, p<0.001; 11.15 ± 2.90 vs.7.77 ± 2.31, p = 0.006 and 2.57 ± 0.48 vs. 1.73 ± 0.59, p = 0.003) (**Table 2**). None of the other parameters showed a significant difference between negative and positive PD-L1 expression.

**Table 2:** Parameters calculated from ^18^F-FAPI-04 PET/CT scans.

Parameters	All patients (n=24)	Negative expression (n=17)	Positive expression (n=7)	P-value
TBR_blood_	9.49 ± 0.56	9.08 ± 2.39	10.49 ± 3.52	0.265
TBR_liver_	9.83 ± 0.84	8.84 ± 3.35	12.23 ± 4.99	0.064
TBR_bone_	9.76 ± 1.00	9.15 ± 4.17	11.22 ± 6.51	0.360
SUVmax*	11.64 ± 0.74	10.61 ± 2.77	14.13 ± 4.41	0.027
SUVmean*	6.59 ± 0.44	5.74 ± 1.60	8.67 ± 1.97	<0.001
SUVpeak*	8.76 ± 0.59	7.77 ± 2.31	11.15 ± 2.90	0.006
SUVsd*	1.98 ± 0.14	1.73 ± 0.59	2.57 ± 0.48	0.003
MTV (cm^3^)	26.55 ± 3.64	18.55 (14.18,37.01)	23.46 (10.46,51.52)	0.930
TLF (g)	182.54 ± 31.73	279.52 ± 246.98	142.62 ± 77.82	0.197

*P < 0.05.

### 3.3 The ability of ^18^F-FAPI-04 PET/CT parameters to predict PD-L1 expression

**Figure 3:**
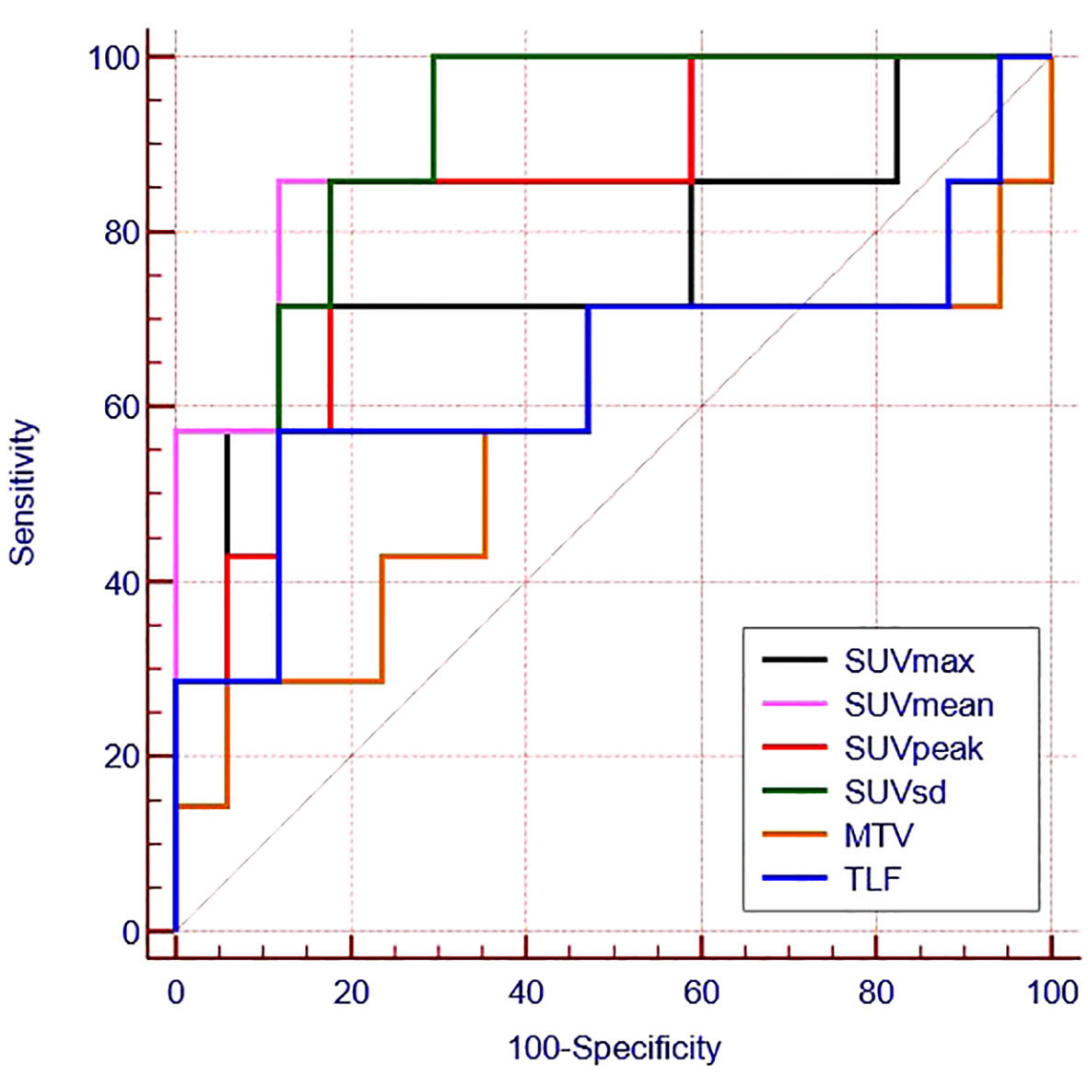
Receiver operating characteristic curves for the ability of 18F -FAPI-04 PET/CT parameters to predict PD-L1 expression.

ROC curves were generated to evaluate the predictive accuracy of ^18^F-FAPI-04 PET/CT parameters for identifying negative and positive expression patients (**Table 3**; **Figure 3**). The AUC value for SUVmean (AUC = 0.882) was higher than those for SUVsd (AUC =0.874), SUVpeak (AUC =0.840) and SUVmax (AUC=0.765) (**Table 3**), while the AUC values for all four parameters were significant (p = 0.004, p = 0.005, p = 0.010 and p = 0.045, respectively) (**Table 3**). The cutoff values for SUVmean, SUVsd, SUVpeak and SUVmax, based on the Youden indexes, were 7.38, 1.90, 9.67 and 13.71, respectively (**Table 3**).

**Table 3:** Areas under the curve for the ability of ^18^F-FAPI-04 PET/CT parameters to predict PD-L1 expression.

Parameters	AUC	Threshold	p	95%CI		Sensitivity	Specificity
				Lower bound	Upper bound		
SUVmax*	0.765	>13.71	0.045	0.520	1.000	71.43	88.24
SUVmean*	0.882	>7.38	0.004	0.718	1.000	85.71	88.24
SUVpeak*	0.840	>9.67	0.010	0.665	1.000	85.71	82.35
SUVsd*	0.874	>1.90	0.005	0.733	1.000	100	80.59
MTV	0.563	>18.55	0.634	0.269	0.857	71.43	52.94
TLF	0.639	>231.98	0.295	0.344	0.933	57.14	88.24
TBR_blood_	0.655	>12.848	0.290	0.436	0.835	42.86	100.00
TBR_liver_	0.706	>12.32	0.124	0.487	0.872	57.14	88.24
TBR_bone_	0.588	>16.685	0.548	0.371	0.783	28.57	100.00

*P < 0.05.

### 3.4 Correlations between ^18^F-FAPI-04 PET/CT parameters and PD-L1 expression

We found a moderate correlation between SUVsd, SUVean, SUVPeak, SUVmax and PD-L1 expression (rs=0.584,p=0.003; rs=0.571,p=0.004; rs=0.511,p=0.011; rs=0.462,p=0.024, respectively). The correlation between ^18^F-FAPI-04PET/CT biomarkers extracted from tumor lesions and PD-L1 expression is shown in **Table 4**.

**Table 4:** Correlations between ^18^F-FAPI-04 PET/CT parameters and PD-L1 expression.

^18^F-FAPI-04 PET/CT Parameters										
PD-L1 expression		SUVmax*	SUVmean*	SUVpeak*	SUVsd*	MTV	TLF	TBR_blood_	TBR_liver_	TBR_bone_
	rs	0.462	0.571	0.511	0.584	-0.016	0.012	0.205	0.270	0.092
	p	0.024	0.004	0.011	0.003	0.941	0.956	0.337	0.203	0.671

*P < 0.05.

### 3.5 Associations between ^18^F-FAPI-04 PET/CT parameters, clinical features and PD-L1 expression

According to univariate logistic regression analyses, SUVmean (p = 0.026), SUVpeak (p = 0.031), and SUVsd (p = 0.024) were independently associated with the PD-L1 expression in LA-ESCC patients (**Table 5**). Due to the moderate positive correlation among SUVmean, SUVsd and SUVpeak, we only included SUVsd (the largest correlation coefficient) and TBRliver (p = 0.059) in the multivariate logistic analysis. Finally, only SUVsd (p = 0.035) was an optimum predictor of PD-L1 expression in these patients (**Table 5**).

**Table 5:** Univariate and multivariate logistic regression analyses of ^18^F-FAPI-04 PET/CT parameters and clinical factors for predicting PD-L1 expression.

Factor	Univariate analysis		Multivariate analysis	
	OR (95% CI)	p-value	OR (95% CI)	p-value
Differentiation degree	1.20 (0.13-11.05)	0.998	–	–
Location	6.0 (0.52-69.75)	0.152	–	–
SUVmax	1.38 (0.99-1.92)	0.051	–	–
SUVmean *	3.10 (1.14-8.41)	0.026	–	–
SUVpeak *	1.74 (1.05-2.87)	0.031	–	–
SUVsd *	16.93 (1.45-19.87)	0.024	3.182 (1.085-9.334)	0.035
MTV	1.02 (0.97-1.07)	0.386	–	–
TLF	1.00 (1.00-1.02)	0.114	–	–
TBR_blood_	1.219 (0.865-1.718)	0.257	–	–
TBR_liver_	1.257 (0.966-1.635)	0.059	0.972 (0.646-1.462)	0.892
TBR_bone_	1.092 (0.908-1.313)	0.348	–	–

*P < 0.05.-,This parameter was not multivariate analyzed.

## 4 Discussion

ESCC is the most common type of esophageal cancer, and about 46.8% of ESCC showed positive PD-L1 expression (19). PD-L1 expression in ESCC is an indicator for immunotherapy and a potential prognostic marker for untreated ESCC patients (20, 21). ^68^Ga-FAPI-04 PET/CT can not only better display the primary tumor and regional lymph nodes, but also the high uptake rate and low background activity of esophageal cancer to facilitate accurate delineation of the target area (22). Therefore, whether parameters from ^18^F/^68^Ga-FAPI-04 PET/CT scans can predict PD-L1 expression in esophageal cancer warrants a prospective study.

In 2019, the immune checkpoint inhibitor pembrolizumab was approved by the Food and Drug Administration (FDA) as a second-line therapy to treat patients with locally advanced or metastatic ESCC whose tumors are positively expressing PD-L1 (Combined Positive Score [CPS] ≥10) (22). Therefore, we chose CPS ≥10 as PD-L1 positive expression in our study and investigated PD-L1 expression correlation with parameters of ^18^F- FAPI-04 PET/CT and clinicopathological characteristics in ESCC.

^18^F-FDG PET/CT can predict tumor microenvironment and PD-L1 expression in many tumors (14, 23). It has been reported that ^18^F-FDG PET/CT can provide metabolic information on tumor immune microenvironment in breast cancer and clear cell renal cell carcinoma (23, 24). Many literatures reported that SUVmax of ^18^F-FDG PET/CT could predict PD-L1 expression in lung adenocarcinomas and squamous cell carcinomas (25, 26). Meanwhile, SUVmax could predict PD-L1 status in cervical cancer (27). These all revealed that the tumor lesion FDG activity (glucose activity) was mainly associated with PD-L1 positive expression.

In our study, the results suggest that specific parameters derived from ^18^F-FAPI-04 PET/CT scans, particularly SUVmean, SUVpeak, and SUVsd were associated with predicting PD-L1 expression of ESCC. By multivariable logistic regression analysis, SUVsd was an optimum predictor for PD-L1 positive expression in ESCC. SUVsd could reflect intratumoral heterogeneity (28–31). The tumor microenvironment can promote the heterogeneity of tumors, including fibroblasts, vascular and immune cells, and the extracellular matrix (32, 33), which also impacts the PD-L1 expression. Thus, different from the correlation between PD-L1 expression and tumor glucose activity (FDG activity) in ^18^F-FDG PET/CT imaging, SUVsd was an optimal predictor of PD-L1 positive expression in ^18^F-FAPI-04 PET/CT imaging, which may be related to its can reflect tumor microenvironment.

The main limitations of the present study include its single-center design and the relatively small sample size. Further large-scale, multi-center clinical studies are needed to confirm our findings before their clinical application. Additionally, many antibodies in PD-L1 expression detection, such as 22C3, SP263, SP142, etc. We use 22C3 for PD-L1 expression detection in our hospital, and the correlation between other methods for PD-L1 expression and ^18^F-FAPI-04 PET/CT parameters needs further study. In addition, probably due to the short follow-up period or small sample size, the ^18^F-FAPI-04 PET/CT parameters could not predict the prognosis of the patients, and we will continue to study this topic.

## 5 Conclusion

^18^F-FAPI-04 PET/CT parameters, including SUVmean, SUVpeak, and SUVsd, were associated with PD-L1 expression in patients with LA-ESCC, and thus, SUVsd was an optimal predict for PD-L1 positive expression, which could help to explore the existence of immune checkpoints and select ESCC candidates for immunotherapy.

## Data availability statement

The original contributions presented in the study are included in the article/supplementary material. Further inquiries can be directed to the corresponding author.

## Ethics statement

The studies involving humans were approved by Tumour Hospital of Shandong First Medical University. The studies were conducted in accordance with the local legislation and institutional requirements. The participants provided their written informed consent to participate in this study.

## Author contributions

YZ: Writing – original draft, Data curation. JR: Software, Writing – original draft, Writing – review & editing.
